# Mechanistic Study on Artificial Stabilization of Lithium Metal Anode via Thermal Pyrolysis of Ammonium Fluoride in Lithium Metal Batteries

**DOI:** 10.1021/acsami.3c17559

**Published:** 2024-04-01

**Authors:** Bereket Woldegbreal Taklu, Wei-Nien Su, Jeng-Chian Chiou, Chia-Yu Chang, Yosef Nikodimos, Keseven Lakshmanan, Teklay Mezgebe Hagos, Gashahun Gobena Serbessa, Gidey Bahre Desta, Teshager Mekonnen Tekaligne, Shadab Ali Ahmed, Sheng-Chiang Yang, She-Huang Wu, Bing Joe Hwang

**Affiliations:** † Nano-Electrochemistry Laboratory, Graduate Institute of Applied Science and Technology, National Taiwan University of Science and Technology, Taipei 106, Taiwan; ‡ Nano-Electrochemistry Laboratory, Department of Chemical Engineering, National Taiwan University of Science and Technology, Taipei 106, Taiwan; § Sustainable Electrochemical Energy Development (SEED) Center, National Taiwan University of Science and Technology, Taipei 106, Taiwan; ∥ Battery Research Center of Green Energy, Ming-Chi University of Technology, New Taipei City 24301, Taiwan; ⊥ National Synchrotron Radiation Research Center (NSRRC), Hsin-Chu 30076, Taiwan

**Keywords:** gas treatment, multiple SEI formation, thermal pyrolysis, multilayered protection, cost

## Abstract

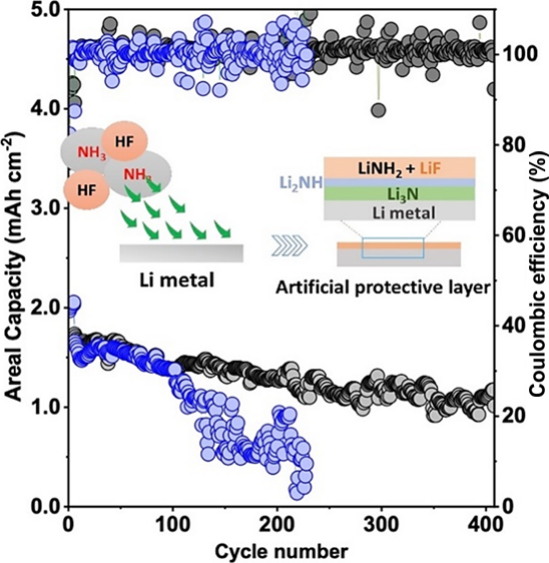

The use of the “Holy Grail” lithium metal anode is pivotal to achieve superior energy density. However, the practice of a lithium metal anode faces practical challenges due to the thermodynamic instability of lithium metal and dendrite growth. Herein, an artificial stabilization of lithium metal was carried out via the thermal pyrolysis of the NH_4_F salt, which generates HF(g) and NH_3_(g). An exposure of lithium metal to the generated gas induces a spontaneous reaction that forms multiple solid electrolyte interface (SEI) components, such as LiF, Li_3_N, Li_2_NH, LiNH_2_, and LiH, from a single salt. The artificially multilayered protection on lithium metal (AF-Li) sustains stable lithium stripping/plating. It suppresses the Li dendrite under the Li||Li symmetric cell. The half-cell Li||Cu and Li||MCMB systems depicted the attributions of the protective layer. We demonstrate that the desirable protective layer in AF-Li exhibited remarkable capacity retention (CR) results. LiFePO_4_ (LFP) showed a CR of 90.6% at 0.5 mA cm^–2^ after 280 cycles, and LiNi_0.5_Mn_0.3_Co_0.2_O_2_ (NCM523) showed 58.7% at 3 mA cm^–2^ after 410 cycles. Formulating the multilayered protection, with the simultaneous formation of multiple SEI components in a facile and cost-effective approach from NH_4_F as a single salt, made the system competent.

## 1 Introduction

The pioneer commercialized Li-ion batteries allowed for the noteworthy advancement of the electric device market and was honored with the Noble Chemistry Prize in 2019.^1,2^ The intercalation–deintercalation in graphite anodes is attributed to its high reversibility, though it has a low energy density of 372 mAh g^–1^.^3,4^ Using lithium metal anode with the highest theoretical capacity (3860 mAh g^–1^) and low electrochemical potential (−3.04 versus the standard hydrogen electrode) is an ultimate choice to develop higher energy density rechargeable batteries.^5−9^ However, the lithium metal anode has an intrinsic issue related to unfavorable dendrite growth, safety, internal short-circuiting, and electrode–electrolyte reactions during operation, which hinders the practical use of lithium metal batteries. Its reactivity drives the electrochemical decomposition of organic liquid electrolytes.^10^ The solid electrolyte interface (SEI) formation at Li metal anodes is uncontrolled and prone to crack accompanied by volume expansion.^6,10,11^ To mitigate parasitic reactions and monitor the lithium flux, a promising strategy of electrolyte additive^12,13^ including alloy formation^14^ is employed to stabilize deposited lithium.^15−20^ The most effective approach is artificially stabilizing the lithium metal anode via gas treatment. Vaporization of iodine on lithium metal to form LiI^21^ and even on solid electrolytes as an additive suggests an effective way to stabilize lithium metal.^22,23^ Other practical approaches include direct N_2_(g) and SO_2_(g) gas treatment of lithium metal and directly on liquid electrolyte, which induces uniformity and stabilizes electrochemical Li plating/stripping attributed to Li_3_N^24^ and inorganic Li_2_S_2_O_4_ formation,^25^ respectively. Vaporized sulfur was also employed to create an artificial Li_2_S layer as a major SEI component with high uniformity and ionic conductivity.^26^ Freon R-134a (1,1,1,2-tetrafluoromethane) as a gas phase reagent is also used to generate lithium fluoride (LiF) upon reaction with lithium metal and effectively mitigates parasitic reactions.^27^

Herein, we present the artificial passivation of lithium metal via the thermal pyrolysis of ammonium fluoride (NH_4_F) as a single salt to generate multiple SEI components. To the best of our knowledge, this approach presents the formulation of artificial multilayered protection to form multiple SEI components simultaneously in a facile and cost-effective way. NH_4_F is an ionic compound only soluble in polar solvents and is rarely used for lithium metal batteries. The protective layer on the lithium metal anode mitigates dendrite growth, polarization, and drastic drops in Coulombic efficiency (CE) under aggressive carbonate electrolyte (1 M LiPF_6_ EC/DEC (1:1 v/v)). The superior electrochemical performance was achieved under the AF-Li||NCM and AF-Li||LFP systems. Advanced characterization tools such as X-ray diffraction (XRD), Raman spectroscopy, and depth profile X-ray photoelectron spectroscopy (depth-XPS) analysis were employed for comprehensive analysis of the composition of different horizons of the multilayered artificial protective layer.

## 2 Experimental Section

### 2.1 Preparation of Materials and Characterization

The thermal pyrolysis of NH_4_F (99.99% Sigma Aldrich) was performed at 180 °C under a sealed container for 2 h, as shown in Figure S1. The pretreated fresh lithium foil, exposed to generated gas to obtain treated lithium metal was then characterized by XRD, Raman spectroscopy, scanning electron microscopy (SEM), energy-dispersive spectroscopy, and X-ray photoelectron spectroscopy (XPS). A commercial electrolyte, 1 M LiPF_6_ in an ethylene carbonate/diethyl carbonate (EC/DEC 1:1 v/v) solution, was received from Sigma-Aldrich. The cathode materials used were LiFePO_4_ (LFP, ∼2 mAh cm^–2^), LiNi_1/3_Co_1/3_Mn_1/3_O_2_ (NCM111, ∼2 mAh cm^–2^), and LiNi_0.5_Mn_0.3_Co_0.2_O_2_ (NCM523, ∼2 mAh cm^–2^), supplied by Advanced Lithium Electrochemistry Co., Ltd. (Aleees), Taiwan. The cathode NCM523 and LFP were coated on Al-foil and mesocarbon microbeads (MCMB) on Cu substrate coated on both sides. The active material on one side was then removed via N-methyl-2-pyrrolidone (NMP, 99%, Aldrich) solvent and punched into a disc (φ = 13 mm, cathode) and (φ = 16 mm, MCMB). The discs were then vacuum-dried at 80 °C overnight, transferred, and processed in an argon-filled glovebox (H_2_O < 0.1 and O_2_ < 0.1).

### 2.2 Battery Assembly and Electrochemical Measurements

Cell configurations of Li||Li, Li||Cu, Li||MCMB, Li||LFP, Li||NCM111, and Li||NCM523 were used to evaluate the effect of the protective layer on lithium metal in terms of the electrochemical performance of the battery. Bare lithium (B-Li) and artificially protected lithium (AF-Li) metal (φ = 16 mm) were used as the anode. A 70-μL commercial carbonate electrolyte of 1 M LiPF_6_-EC/DEC (1:1 v/v) was used. Celgard 2325 (φ = 19 mm) was used as the separator. AC impedance coupled with cyclic voltammetric (CV) measurement was recorded on a Biologic SAS. Electrochemical impedance spectroscopy (EIS) measurements were conducted within a frequency range of 1–10 mHz under a voltage amplitude of 10 mV.

### 2.3 Computational Estimation of Formation Energy (Δ*E*_f_)

DFT-D3 calculations were employed to support the proposed reaction mechanism and determine the formation energy for reaction energies for each species participating in the reactions.^28,29^ The computation with D3 correction was performed for energy calculations on the optimized minimum energy structure, initially obtained without correction.^3^ Subsequently, the energy change (Δ*E*) of the reaction was evaluated by employing a general formula.
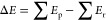


## 3 Results and Discussion

### 3.1 Reaction Mechanism and Compositional Analysis

A promising artificial protective layer via the gas treatment of lithium metal from thermal pyrolysis of NH_4_F was undertaken in an in-house sealed container, as shown in Figure S1. Solid NH_4_F, upon heating (180 °C, 2 h), transformed into a liquid phase with the formation of gaseous species as indicated in reaction 1
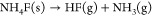
1

**Figure 1:**
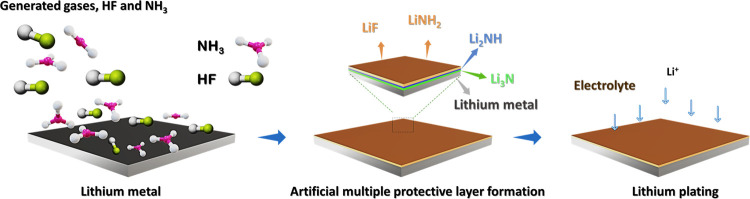
Schematic illustration for the formation of an artificial multilayered protection via gas treatment and its effect on lithium plating.

The polished and rolled lithium metal was then exposed to generated gas, HF(g) and NH_3_(g), for 2 h to obtain treated lithium metal. The scheme in Figure 1 demonstrates the formation of the passivation layer over exposure to NH_3_ and HF gas. The formed artificial SEI avoids electrolyte decomposition, corrosion, and dead lithium metal formations. Interestingly, we proposed a mechanism for the formation of LiF(s), Li_3_N(s), and LiH(s) upon reaction with lithium metal. The material characterization via XRD and Raman measurement of the treated lithium metal surface indicates the formation of other species, such as LiNH_2_ and Li_2_NH.

The proposed reaction mechanism using DFT-D3 calculations, where Δ*E*_f_ = +ve (positive value) implies unfavorable reaction, while Δ*E*_f_ = −ve (negative value) indicates the highly thermodynamically relevant reaction. The detailed stepwise reaction mechanism in Table 1, from (2) to (8) outlines the formation energy (ΔE_f_), encompassing reactions involving HF(g) and NH_3_(g) with the freshly exposed lithium metal surface.

**Table 1:** Stepwise Chemical Reaction and Formation Energy for Each Reaction

stepwise reactions	Δ*E*_f_ (kJ mol^–1^)	stepwise reactions	Δ*E*_f_ (kJ mol^–1^)
2NH_3_(g)+ 6Li → 2Li_3_N(s) + 3H_2_ (g)..................(2)	–10.64	Li_2_NH(s) + H_2_(g) ↔ LiNH_2_(s)+ LiH(s) ............(6)	2.04
HF(g) + Li → LiF(s) + H_2_(g) ..............................(3)	–25.30	2LiNH_2_(s) → Li_2_NH (s) + NH_3_(g).....................(7)	–0.50
H_2_(g) + 2Li → 2 LiH(s) ........................................(4)	–20.45	NH_3_(g) + LiH(s)→LiNH_2_(s)+ H_2_(g)................(8)	–22.22
Li_3_N(s) + 2H_2_ (g)→ Li_2_NH(s) + LiH(s) .............(5)	–5.18		

**Figure 2:**
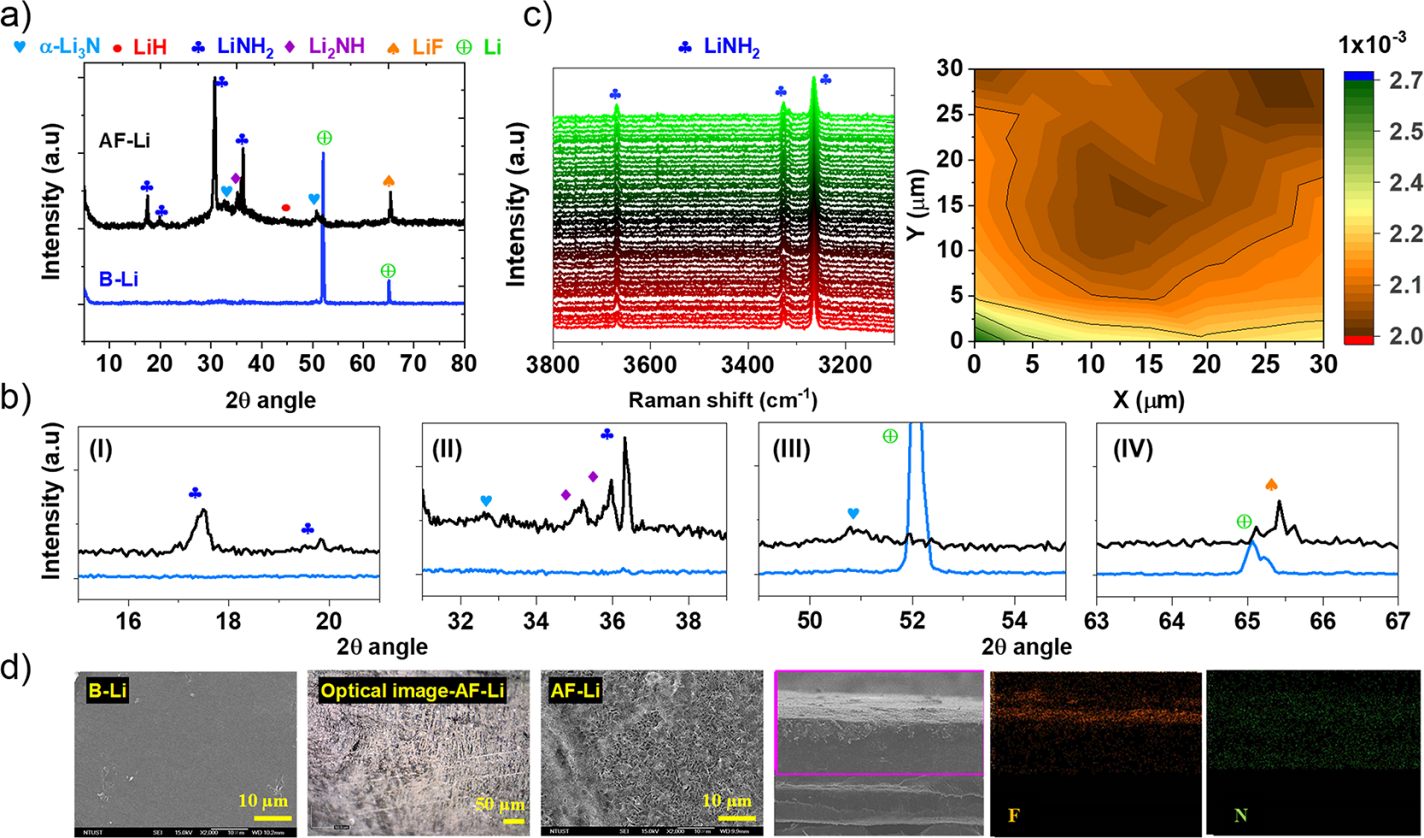
Material characterization of treated lithium metal, AF-Li, and bare lithium metal, B-Li. (a) XRD patterns for AF-Li and B-Li metal anode. (b) Insets of XRD plots at different regions as (I), (II), (III), and (IV). (c) Raman spectra and mapping of AF-Li metal surfaces. (d) SEM images of B-Li; optical image and cross-sectional view of AF-Li.

The reactions indicated in (2) and (3) result in Li_3_N(s) and LiF(s) formation. The computed formation energy, Δ*E*_f_ (in kJ mol^–1^), indicates thermodynamic favorability. LiF(s) product formed from the reaction with a formation energy of Δ*E*_f_ = −25.30 kJ mol^–1^, thermodynamically more favorable than Li_3_N formation (Δ*E*_f_ = −10.64 kJ mol^–1^). However, still the formation of Li_3_N is also a dominating reaction which is also confirmed with the formation of brown coloration on the lithium metal surface upon the reaction, as shown in Figure S2. A recent report on the use of nitrogen gas, N_2_(g), in an argon atmosphere (10% v/v) upon exposure to lithium metal showed the formation of Li_3_N (brown color) on the lithium surface.^24^ The reaction byproduct, hydrogen gas, H_2_(g), generated from reaction (2) and reaction (3) then further reacts with lithium metal to form LiH(s) as indicated in reaction (4) with formation energy of Δ*E*_f_ = −20.45 kJ mol^–1^. The presence of excess H_2_(g) and NH_3_(g) in the system initiates stepwise transformation to monohydrogenation, Li_2_NH with formation energy, Δ*E*_f_ of −5.18 kJ mol^–1^, and dissociation reaction (Δ*E*_f_ = −0.50 kJ mol^–1^) from LiNH_2_(s) in reaction (5) and (7) respectively. However, the formed Li_2_NH is thermodynamically stable with a positive formation energy, Δ*E*_f_ = 2.04 kJ mol^–1^. The dihydrogenation product, LiNH_2_ with high formation energy, Δ*E*_f_, of −22.22 kJ mol^–1^ was formed between NH_3_ and LiH as indicated in the stepwise reaction (8).^30,31^ The formed LiH(s) from reactions (4) and (5) were subsequently consumed during the progress of the reaction, as seen in reaction (8).^32−36^ To elucidate the formed species, XRD was undertaken via an airtight XRD sample holder on the lithium metal surface. The simultaneous formation of Li_2_NH(s) and LiNH_2_(s) with varied peak intensities was identified. Principal peaks at around 17.5°, 30.8°, and 36.3° 2θ angles belong to detected LiNH_2_. The peak with a relative intensity associated with Li_2_NH formation appeared at 35.9° 2θ angle, which is dominated by LiNH_2_, as indicated in reactions (5) and (8).^32,34,37−39^ In addition, a characteristic peak at around 65.5° 2θ belonging to LiF formation was also detected. It is observed that the formed LiH has insignificant intensity at 44.3° 2θ angle as a result of subsequent reaction as shown in Figure 2a,b I–IV. The formed LiH acts as an intermediate species formed and consumed by the progress of the reaction rather than being a principal product. Similarly, the less intense peak was identified at around 32.6° and 50.8° 2θ angle belongs to α-Li_3_N formation mainly related to subsequent consumption and transformation to Li_2_NH as indicated from reaction (5). Raman spectra and Raman mapping were carried out to confirm further the species formed for lithium AF-Li, as indicated in Figure 2c. The principal peak correspondingto LiNH_2_ appeared as a dominant peak at around 3265 and 3225 cm^–1^ on the treated lithium metal surface, similar to the reported ball mill-based formation of LiNH_2_ from Li_3_N and LiH.^32^ These peaks were not detected in bare lithium metal, as shown in Figure S3a. After treatment, the Raman mapping also indicates the uniform distribution of the protective layer on the lithium metal surface. The cross-sectional SEM image and EDX elemental mapping of AF-Li demonstrate the compositional uniformity of nitrogen and fluorine on the treated lithium metal surface. In addition, the color mapping of the optical image of treated lithium metal also shows the extent of coverage of the coating material on the lithium metal surface, as indicated in Figure S3b I–IV.

**Figure 3:**
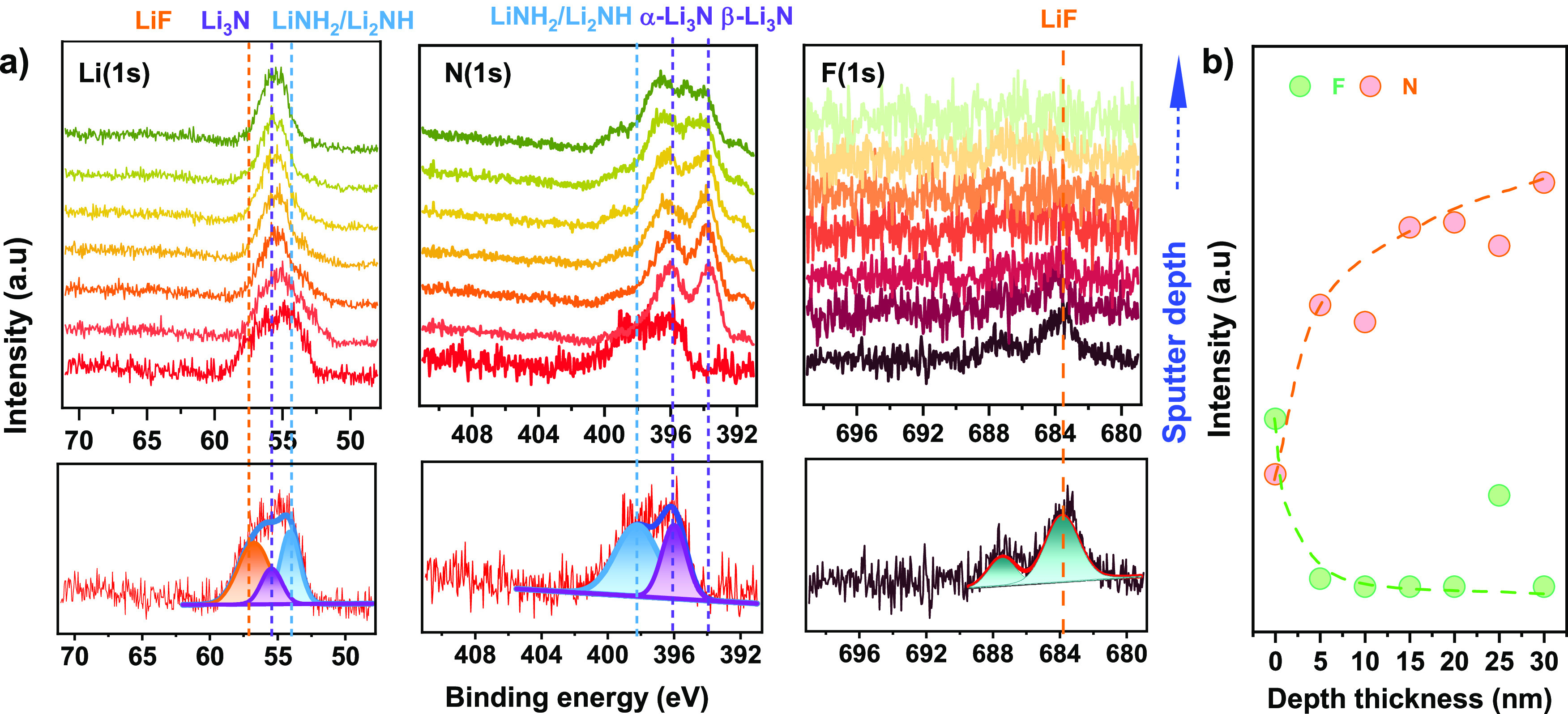
Compositional evolution of treated lithium metal, AF-Li, as a function of the sputtering time. (a) Depth profile XPS measurements of AF-Li over Li(1s), N(1s), and F(1s) core. (b) Intensity of elemental contents as a function of depth thickness for F and N.

To further examine the formed artificial SEI components, the XPS depth profile was measured for Li(1s), F(1s), and N(1s) to scrutinize the elemental content and its relative intensity, as shown in Figure 3. The signals were collected after 30 min etching time interval with the Ar500+ cluster ion source. An elemental compositional evolution over the sputtering depth was recorded for treated lithium metal, AF-Li. It is indicated that LiF, Li_2_NH, and LiNH_2_ dominate the outer surface of the protective passivation layer due to the continuous transformation of intermediate products. The constant exposure to reactive gas initiates the transformation of the hydrogenation reaction of nitride, Li_3_N, which leads to the formation of amide species. However, as the sputtering depth increases, the peak belonging to Li_3_N (at 55.45 and 395.2 eV) becomes more evident and intense with decreased intensity of LiNH_2_ (399.4 eV), as indicated in Figure 3. Wood et al.^40^ also presented the hydrogenation of Li_3_N, resulting in the formation of a multilayered structure. The reacted Li_3_N in the presence of H_2_ results in Li_2_NH from outside, and conversion of NH_3_ into LiNH_2_ in the presence of LiH as a shell, which makes the more intense spectra both on the XRD and Raman measurement. On the other hand, lithium metal’s strong exposure to HF results in LiF formation. A multilayer structure was formed where Li_2_NH and LiNH_2_ envelop Li_3_N due to constant and full exposure of Li_3_N to H_2_(g) as well as LiH to NH_3_(g) from outside, as indicated in the reactions (5) and (8), as shown in Figure 1. The peak intensity dominates from outside and decreases with sputtering depth, confirming that the lithium metal only reacts on the exposed surface.

### 3.2 Multilayered Lithium Protection and Suppression of Lithium Dendrite

**Figure 4:**
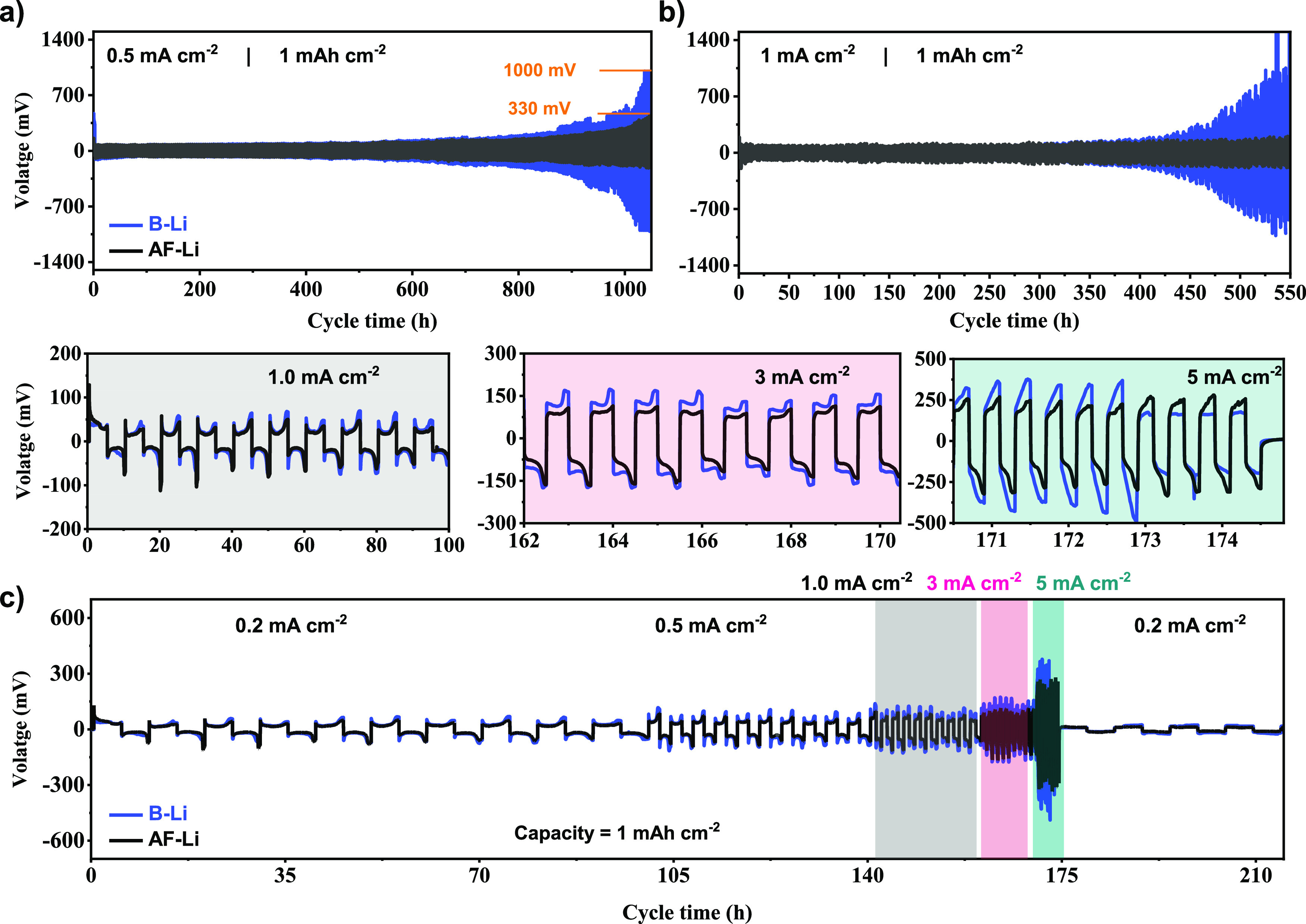
Electrochemical performance of B-Li (blue) and AF-Li (black) under symmetric cells in conventional carbonate electrolyte, 1 M LiPF_6_ EC/DEC (1:1 v/v). (a) and (b) Symmetric cells operated at 0.5 and 1 mA cm^–2^ current density with 1 mAh cm^–2^ areal capacity. (c) Critical current density (CCD) of the test of symmetric cells operated at different current densities and its inset operated a fixed areal capacity of 1 mAh cm^–2^.

Li||Li symmetric cell was performed using a commercial electrolyte, 1 M LiPF_6_ EC/DEC EC/DEC (1:1 v/v), to investigate the effect of the artificial protective layer on lithium metal stabilization. A fixed reversible areal capacity of 1 mAh cm^–2^ was used to operate the cell. At 0.5 mA cm^–2^ current density, the protected lithium metal started at a relatively high voltage of 160 mV in contrast to 100 mV in bare lithium metal. The resistance originated from a protective layer and limited wettability at an early stage attributed for voltage increment. Over cycling, the voltage hysteresis in B-Li was increased by 10-fold with voltage fluctuations and reached the cutoff limit of 1000 mV after 1050 h (525 cycles) caused by continuous electrolyte decomposition and impedance growth at the lithium/electrolyte interface. However, artificial SEI in AF-Li stabilized the interface. It led to the decrease in the voltage to 130 mV after 800 h (400 cycles), ascribed to the effective protection of lithium metal. It later increased to 330 mV after 1050 h, as indicated in Figure 4a. Likewise, severe overpotential growth and voltage hysteresis were found in B-Li metal from continuous growth in internal resistance at 1 mA cm^–2^ high current density. The treated lithium metal AF-Li showed outstanding stability over multiple plating/stripping with insignificant polarization. It ran for more than 550 h, as depicted in Figure 4b.

To further elucidate tolerance to short circuits at high current, critical current density (CCD) at 0.2, 0.5, 1, 3, and 5 mA cm^–2^ was performed under Li||Li symmetric cell. At a lower current density, 0.2 mA cm^–2^ for treated and bare lithium metals shows smooth cyclability with lower overpotential, as indicated in Figure 4c inset from 0 to 100 h. However, as the cycle turns to high current density, a noticeable overpotential increment was observed in bare lithium metal, mainly related to the corrosion of lithium metal in contact with the electrolyte, as depicted in Figure 4c; inset from 162 to 170 h. Interestingly, at high current density, 5 mA cm^–2^, an increase in overpotential coupled with abrupt voltage drops was observed as an indicator for internal short circuits, as shown in Figure 4c (inset from 170 to 175 h). However, the artificial multilayered protection on the lithium metal results in abatement in chemical instability and reduces overpotential due to a stable SEI layer on the Li metal electrode.

**Figure 5:**
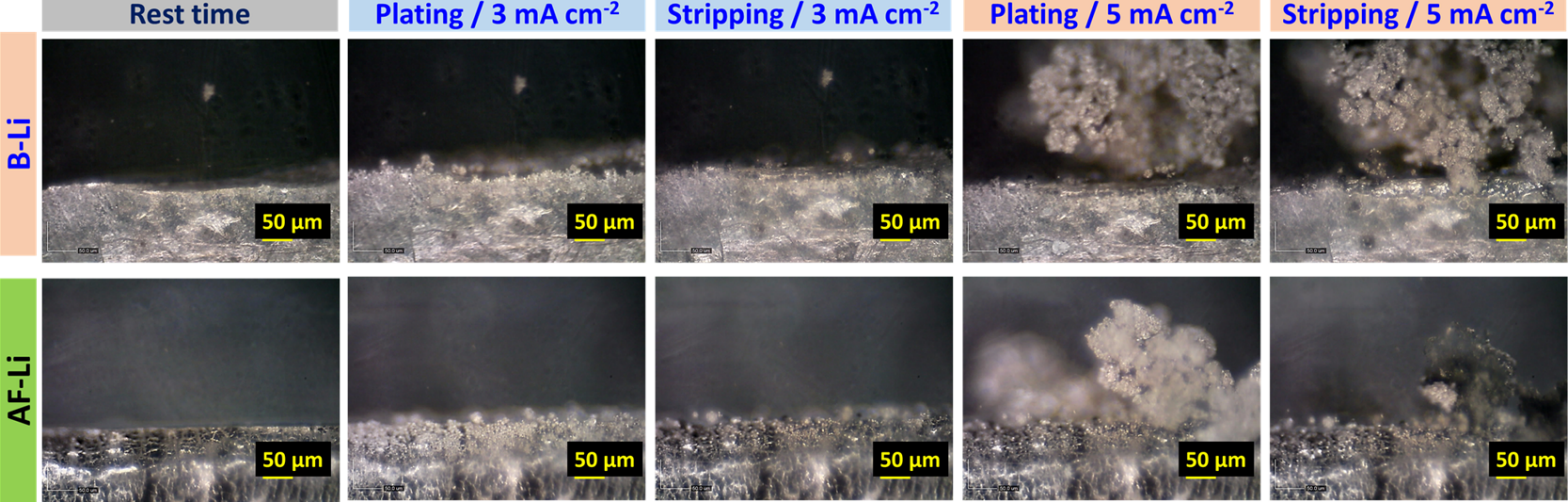
Operando OM observation on plating/stripping phenomena in symmetric cells operated at 3 and 5 mA cm^–2^ current density for B-Li and AF-Li metal system in 1 M LiPF_6_ EC/DEC (1:1 v/v) electrolyte.

To visualize the lithium metal reversibility and dead lithium metal formation, an operando optical microscopy (operando OM) measurement was performed at 3 and 5 mA cm^–2^ current density under a symmetric cell configuration as shown in Figure 5. At 3 mA cm^–2^ current density on the plating step, B-Li showed mossy dendritic lithium growth compared to AF-Li. However, the dendrite growth is more evident at high current density, 5 mA cm^–2^ in both cases, but more violent in the B-Li metal. The dendritic lithium in B-Li on the stripping step did not return back, stood as mossy lithium, which facilitates the fast short circuit, and also agreed with the CCD test in Figure 4 inset and Figure S4a–d inset. In the AF-Li, the mossy dendritic lithium formed and was then entirely stripped with some formed transparent lithium metal from SEI formation. The AF-Li metal showed outstanding lithium reversibility even at an extremely high current density, 5 mA cm^–2^, without short circuit formations. The voltage polarizations in AF-Li at 5 mA cm^–2^ are mainly related to the mossy lithium formation in the plating step, as indicated in Figure 4c.

**Figure 6:**
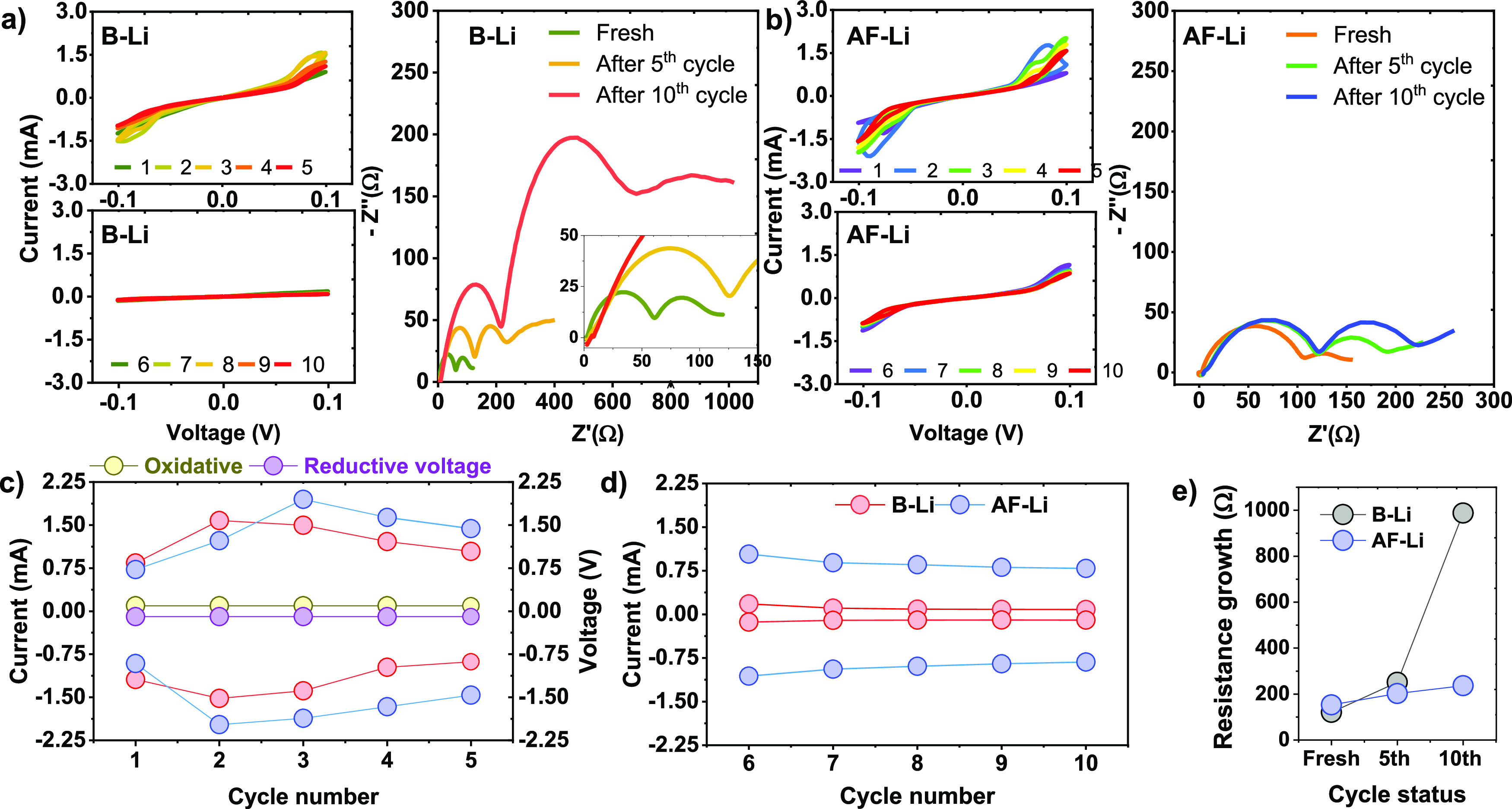
AC impedance integrated CV measurements for the symmetric cell under B-Li metal anode and AF-Li metal anode operated at 0.01 mV s^–1^ scan rate. (a, b) Current–voltage curves for both B-Li and AF-Li metal and their EIS measurements at different cycle numbers. (c, d) Peak current for B-Li and AF-Li at +95.5 mV and −95.5 mV for oxidative and reductive current. (e) EIS growth as a function of cycle number for both B-Li and AF-Li metal.

**Figure 7:**
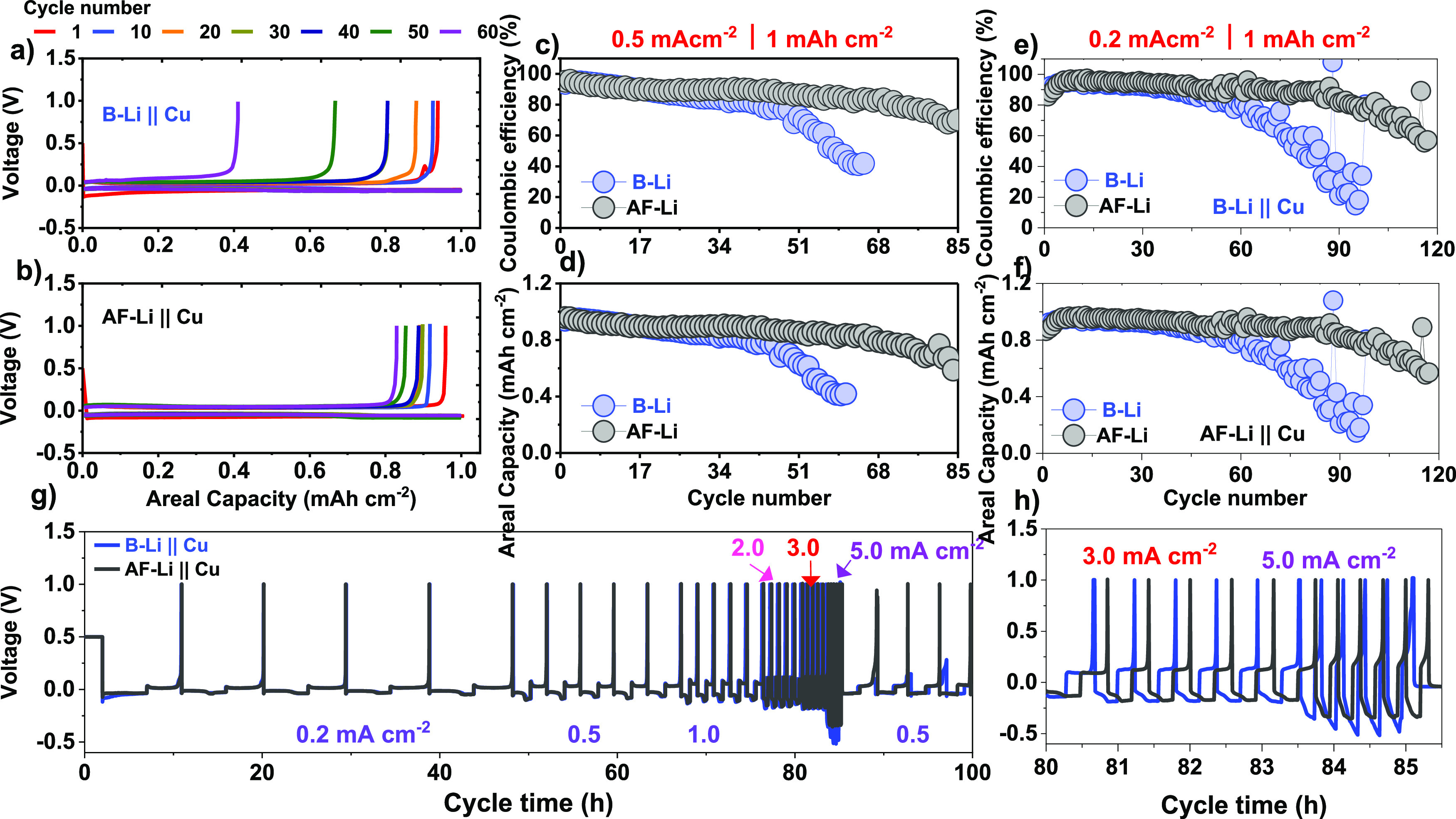
Half-cell electrochemical performance of B-Li and AF-Li at 0.2 and 0.5 mA cm^–2^ current densities. (a) and (b) Voltage profile for B-Li and AF-Li metal anode with 1 mAh cm^–2^ areal capacity operated at 0.5 mA cm^–2^ current density. (c) and (d) are CE and charge capacity of B-Li at 0.5 mA cm^–2^ current density. (e) and (f) are CE of B-Li and AF-Li metal anodes at 0.2 mA cm^–2^ current density. (g) CCD test for half cell, x-Li||Cu (x = B or AF) at fixed areal capacity 1 mAh cm^–2^ and (h) its inset in the range of 80–85.5 h.

**Figure 8:**
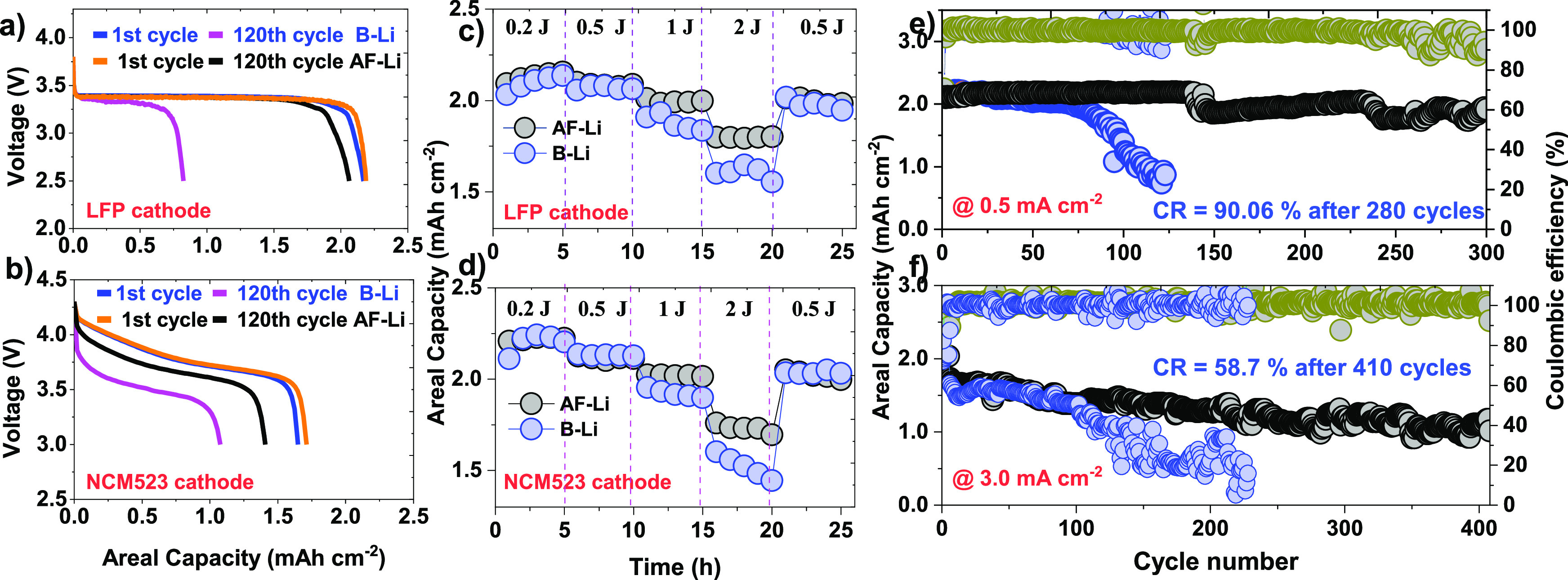
Electrochemical performance of the full cell with LFP||x-Li (x = B or AF) and NCM523||x-Li (x = B or AF) at different operative conditions. (a) and (b) are voltage profiles for LFP and NCM523 cathode for AF-Li and B-Li metal anode at the 1^st^ and 120^th^ cycle. (c) and (d) are rate capability under the LFP and NCM523 cathode coupled with AF-Li and B-Li metal anode. (e) and (f) are the LFP||x-Li (x = B or AF) and NCM523||x-Li (x = B or AF) at 0.5 mA cm^–2^ and 3.0 mA cm^–2^ current density.

Interestingly, the optimistic influence of the protective layer on lithium metal is also evidenced via a CV study coupled with AC impedance measurement, as shown in Figure 6. To elucidate the alteration in the resistance of the Li||Li symmetric cell, fresh state, after the fifth cycle and 10^th^ cycle for both B-Li (Figure 6a) and AF-Li (Figure 6b), was considered. The impedance in B-Li significantly increased by 8.2 times after the fifth cycle; however, for AF-Li, the impedance increased by 1.54 times with reference to the fresh state, respectively. The oxidative and reductive peak current recorded at +95.5 mV and −95.5 mV showed an increase to maximum peak current and later decrease in the current signal for both to 5^th^ cycle. However, the current signal in B-Li is smaller than AF-Li, indicating that the lithium kinetics at AF-Li | electrolyte is enhanced compared to B-Li by some factor, as indicated in Figure 6c, e. For up to the 10^th^ cycle, at early cycles (6^th^ cycle), the oxidative and reductive peak current for AF-Li has a relatively high current and later becomes almost constant, indicating lithium kinetics AF-Li | electrolyte looks uniform and steady. Remarkably, for B-Li, the current signal becomes close to zero, also in agreement with the impedance growth. The lithium kinetics at the B-Li | electrolyte interface is severely affected by the electrolyte decomposition, and the reaction with lithium metal anode ultimately leads to the short circuit and capacity fading, as indicated in Figures 4c, 7, and 8.

### 3.3 Half-Cell Electrochemical Performance with Cu and MCMB Substrates

The half-cell Li||Cu and Li||MCMB performances were used to examine the impact of the protective layer on the lithium metal surface as a function of polarization and CE for B-Li and AF-Li metal. The extent of electrolyte decomposition toward B-Li and AF-Li via Cu and MCMB as a substrate was considered with the use of carbonate electrolyte. The plating/stripping in x-Li||Cu (x = B-Li or AF-Li) cells at 0.2 and 0.5 mA cm^–2^ (capacity 1.0 mAh cm^–2^) is indicated in Figures 7a, b, S5, and S6. The phenomena of intercalation/deintercalation in x-Li||MCMB (x = B-Li or AF-Li) at 1 mA cm^–2^ (capacity 4.0 mAh cm^–2^) investigated for both B-Li and AF-Li are shown in Figures S7, S8, and S10, respectively. Despite the CE being reliant on lithium loss on the Cu side, the decomposition of electrolytes is mainly linked to the extent of the parasitic reaction toward plated lithium metal and the bulk lithium metal anode. At early cycles, regardless of the lithium metal anode type used, x-Li||Cu cells assembled at 0.5 mA cm^–2^ using B-Li and AF-Li hardly showed a significant variation on initial CE (iCE) of about 93.85 and 95.18%, respectively. Nevertheless, the electrolyte’s decomposition during cycling leads to increased resistance due to the creation of a resistive SEI at the lithium interface, occurring on both the copper and bulk lithium metal substrate. The alteration of interface phenomena directly shows from the voltage profile with an overpotential growth and leads to incessant fading over cycles, as shown in Figures 7a,b and S5. In addition, the voltage–time profile of half-cell B-Li||Cu at a 0.5 mA cm^–2^ current density shows polarization before it reaches 200 h. Nevertheless, the treated lithium metal, AF-Li||Cu cell, can run more than 300 h without significant polarizations. An average Coulombic efficiency (ACE) of 78.3% was achieved with B-Li metal as an anode after 60 cycles. Nevertheless, AF-Li delivers ACE of about 89% at the same cycle number, as shown in Figure 7c,d. Furthermore, at a low current density of 0.2 mA cm^–2^, the B-Li||Cu half-cell operated for 840 h with fading of charge capacity coupled with a drop in CE. However, AF-Li||Cu ran for 1100 h with significant improvement over the cycle life and CE, as shown in Figures 7e,f, and S6. The communal effect coming from the electrolyte decomposition on the plated lithium metal and bulk lithium anode (B-Li) leads to overpotential growth together with a lowering in ACE. In this regard, the use of an artificial protective layer on bulk lithium metal reasonably boosts interface stability and mitigates the electrolyte decomposition, which results in a decrease in the overpotential in comparison with the B-Li anode. The use of electrolyte additives mostly influences on simultaneous stabilization of plated lithium metal and bulk lithium metal.^40,41^ Surprisingly, the protection of lithium metal significantly impacts the reversibility and life cycle of batteries irrespective of considering plated lithium metal.

Similarly, the discharge–charge performance of the Li||MCMB half-cell was employed to realize the effect of the artificial protective layer on AF-Li, as indicated in Figure S7. The cell was operated at a current density of 1 mA cm^–2^ with a reversible 4 mAh cm^–2^ capacity. The SEI layer formed in the MCMB electrode is believed to be identical. The iCE values for B-Li and AF-Li have no significant variations, 92.4 and 93.8%, respectively. However, after the 30th cycle, the CE with the AF-Li metal anode is 98.9%, while with the B-Li metal anode is 93.4%. This clearly indicates that the electrolyte decomposition on the bare lithium metal anode is inevitable and induces resistance attributed to limited reversibility and overpotential growth. The B-Li||MCMB cell can only run for 400 h with severe voltage hysteresis and fluctuation, while the AF-Li||MCMB cell operated for more than 750 h, as depicted in Figure S8. Later, the B-Li||MCMB cell showed a roll-over failure after the 54^th^ cycle. The CV coupled with AC impedance measurement was undertaken to examine the cell’s reversibility. Curiously, the protected lithium metal anode under the AF-Li||MCMB cell at the fresh state has EIS values of 114.2 Ω; later after multiple intercalation–deintercalation, the resistance then diminished to 15.7 Ω due to stabilization from artificial SEI formation. The artificial protective layer on the AF-Li lithium metal anode delivers excellent protection, and its reversibility is proven by CV measurement with the MCMB electrode, as presented in Figure S8a,b. In contrast, the bare lithium metal anode, B-Li||MCMB cell, has an impedance of 61.5 Ω in the fresh state and 41.9 Ω after cycling. The CV curve in B-Li||MCMB showed an abrupt drop and disturbance mainly due to resistive interface from electrolyte decomposition at B-Li/electrolyte interface as given in Figure S8c,d. Furthermore, the cell test operated at a high current density of 2 mA cm^–2^/2 mAh cm^–2^, AF-Li||MCMB cell run for more than 850 h with the retention capacity of 98.8% and for B-Li||MCMB 91.6% were obtained after 160 cycles, as indicated in Figure S10a–c. The B-Li||MCMB cell operated for 650 h followed by drastic voltage fluctuation and short circuit formation as indicated in Figure S9a–c and Figure S11.

### 3.4 Electrochemical Performance in Full-Cell Configurations

To demonstrate the reliability of the artificial protective layer on the lithium metal anode, full-cell LFP||x-Li (x = B-Li or AF-Li), and NCM523||x-Li (x = B-Li or AF-Li) were performed at 0.5 mA cm^–2^, and 3 mA cm^–2^ current density, respectively, as shown in Figure 8. The cyclic performances of B-Li and AF-Li with an LFP cathode were operated under a 3.8 V cutoff voltage. The rate capability test for LFP||AF-Li at 0.2 mA cm^–2^ delivers a relatively higher discharge capacity of 2.16 mAh cm^–2^, as compared to 2.13 mAh cm^–2^ in LFP || B-Li. As the current density increases to 2 mA cm^–2^, the B-Li and AF-Li cells showed capacity fading as 1.55 and 1.80 mAh cm^–2^, respectively. This clearly shows the protective layer on the lithium metal, which efficiently mitigates electrolyte decomposition at the electrolyte/anode interface. Moreover, long cycling stability operated at 0.5 mA cm^–2^ current density, with an achieved capacity retention (CR) of 90.6% after 280 cycles in LFP || AF-Li cell as shown in Figure 8a, e. In contrast, the LFP||Li full cells indicated in Figure 8a,e recede before 120 cycles, indicating continuous growth of resistive passivation layer formation and dead lithium at the interface. The artificial protective layer on lithium metal results in substantial resilience to lithium dendrites and favors a robust interface.

Furthermore, the NCM523|| x-Li (x = B or AF) operated at 3 mA cm^–2^; the AF-Li metal anode showed superior performance over the B-Li anode, as shown in Figure 8b, d, f. The CR of 58.7% was achieved at 3 mA cm^–2^ current density after 410 cycles, while the B-Li anode failed before 170 cycles. Interestingly, the CV measurements of NCM523|| x-Li (x = B or AF) cell are shown in Figure S12a–d. The polarization (ΔV) of 1.84 V was recorded in the B-Li anode compared to 1.64 V in the AF-Li metal anode after the 5^th^ cycle. The interface stabilization was also confirmed by CV measurements with lowered impedance after cycling, as indicated by EIS measurement in Figure S12e,f. Also, NCM111 || x-Li (x = B or AF) cell was further employed to study electrochemical performance. The cell performance at 0.2 cm^–2^ current density under AF-Li metal anode delivers a CR of 93.2% after 80 cycles while with B-Li metal 85.2%, as shown in Figure S13. In the rate capability of NCM523|| x-Li (x = B or AF) cell at 0.2 mA cm^–2^, the AF-Li anode delivers a relatively higher discharge capacity of 2.22 mAh cm^–2^, as compared to 2.20 mAh cm^–2^ in B-Li. When the current density increases to 2 mA cm^–2^, the B-Li and AF-Li anode shows a discharge capacity of 1.69 and 1.44 mAh cm^–2^, respectively. The formation of a protective layer with multiple SEI components plays a vital role in superior cycling performance when compared with a bare lithium metal anode.

## 4 Conclusions

In summary, we have designed the simultaneous formulation of multiple SEI components from the thermal pyrolysis of NH_4_F as a single salt. The facial and cost-effective approach used for the artificial protective layer formation makes it suitable for large-scale processing from a less costly percussor, NH_4_F. XRD, Raman, and depth profile XPS studies confirm the formation of multiple SEI components with multilayered protections. The treated Li (AF-Li) metal showed a superior dendrite suppression capability of 5 mA cm^–2^ under low overpotential evolution in the symmetric cell and AF-Li||Cu half-cell. The cell operated at 0.2 and 0.5 mA cm^–2^ current density run for more than 1100 and 300 h, respectively. The protective layer provides excellent protection and reduces the lithium metal reaction vulnerability with the electrolyte. In addition, the treated lithium metal AF-Li with an MCMB substrate at 1 mA cm^–2^ current density also operated for more than 750 h and fully outperformed the B-Li metal anode. A full-cell assembled from the LFP, NCM111, and NCM523 cathode with an AF-Li anode showed superior performance at 0.5, 0.2, and 3 mA cm^–2^ operated for more than 300, 85, and 410 cycles, respectively. These outstanding properties of the protective layer mitigate electrolyte consumption, decomposition, and impedance growth. The present work signifies as a springboard toward the practical use of lithium metal anodes in rechargeable batteries.

## Supporting Information Available

Preparation of materials and synthesis scheme; material characterization, battery assembly, electrochemical measurements, computation for formation energy (Δ*E*_f_), and results; half-cell, x-Li||MCMB and x-Li||Cu, (x = B-Li or AF-Li); cyclic voltammetric measurements for x-Li||MCMB and NCM-523 || x-Li system; and electrochemical performance for NCM-111 || x-Li (x = B or AF) (PDF)
