# Supplementary material for: Mechanistic Study on Artificial Stabilization of Lithium Metal Anode via Thermal Pyrolysis of Ammonium Fluoride in Lithium Metal Batteries
Source: ACS Appl Mater Interfaces. 2024 Apr 1;16(14):17422–31. doi: 10.1021/acsami.3c17559 (PMC11009921; doi:10.1021/acsami.3c17559)
Supplement: Supplementary file 1 — am3c17559_si_001.pdf [file am3c17559_si_001.pdf]

Supporting Information

Mechanistic Study on Artificial Stabilization of Lithium-Metal Anode via Thermal Pyrolysis of Ammonium Fluoride in Lithium Metal Batteries

Bereket Woldegbreal Taklu,[a,c] Wei-Nien Su,*[a,c] Jeng-Chian Chiou,[b] Chia-Yu Chang,[b] Yosef Nikodimos,[b] Keseven Lakshmanan,[a] Teklay Mezgebe Hagos,[b] Gashahun Gobena Serbessa,[b,d] Gidey Bahre Desta,[a] Teshager Mekonnen Tekaligne,[b] Shadab Ali Ahmed,[a] Sheng-Chiang Yang,[b] She-Huang Wu,[a,c] and Bing Joe Hwang*[b,c,e]

[a] Nano-electrochemistry laboratory, Graduate Institute of Applied Science and Technology, National Taiwan University of Science and Technology, Taipei 106, Taiwan

[b] Nano-electrochemistry Laboratory, Department of Chemical Engineering, National Taiwan University of Science and Technology, Taipei 106, Taiwan

[c] Sustainable Electrochemical Energy Development (SEED) Center, National Taiwan University of Science and Technology, Taipei 106, Taiwan

[d] Battery Research Center of Green Energy, Ming-Chi University of Technology, New Taipei City, 24301, Taiwan

[e] National Synchrotron Radiation Research Center (NSRRC), Hsin-Chu, 30076, Taiwan

*Corresponding Authors:

Wei-Nien Su, wsu@mail.ntust.edu.tw

Bing Joe Hwang, bjh@mail.ntust.edu.tw

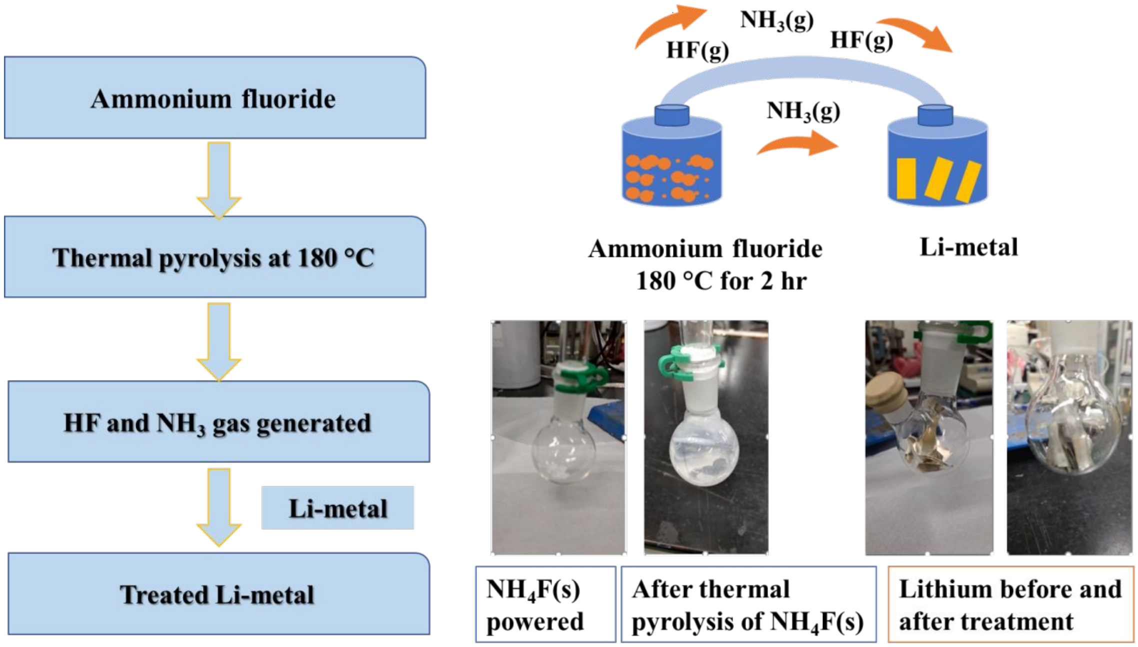

Figure S1. Schematic follow chart and pictorial demonstration of the lithium metal treatment at 180 °C at 2 hr.

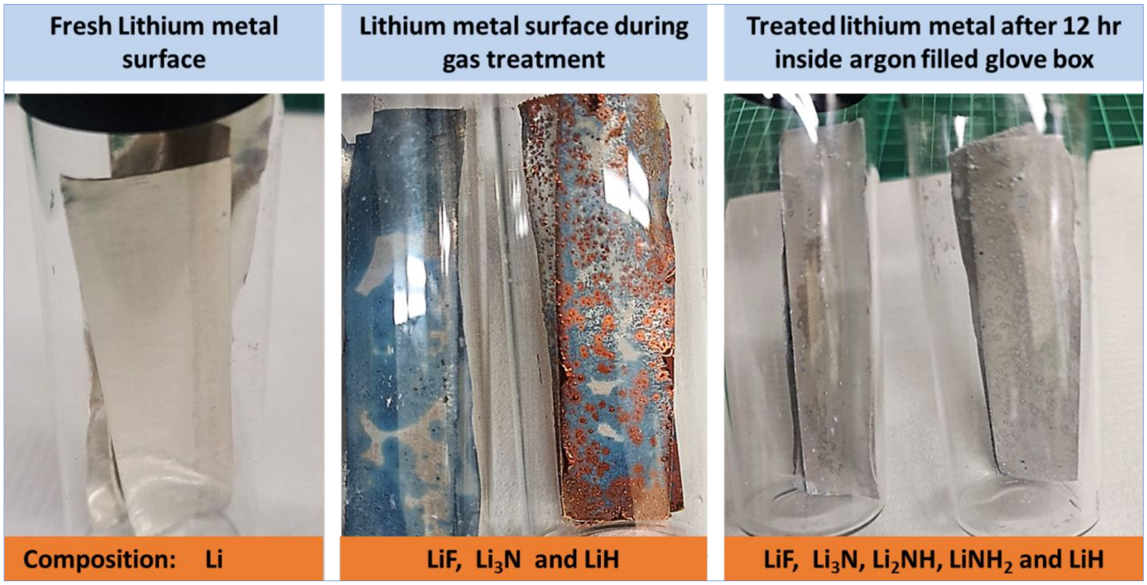

Figure S2. The optical image of fresh lithium metal surface, after gas treatment and after being stored in the glove box for 12 hr respectively. Coloration to deep blue and brow color mainly due to LiF, LiH, and Li_3N formation, further transformation reactions completed overnight lead to powdery grey lithium surface mainly due to Li_2NH, and $LiNH_2$ formation.

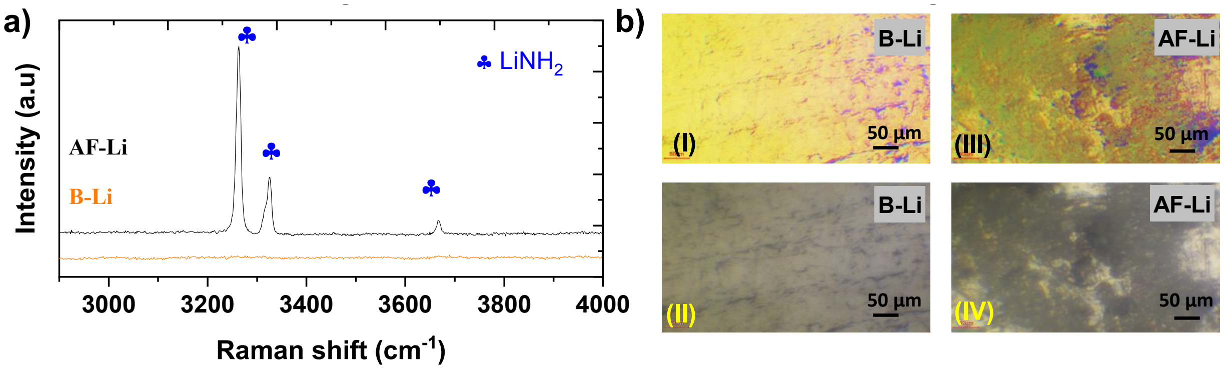

Figure S3. Material characterization of protected lithium metal, AF-Li, and bare lithium metal, B-Li. a) Raman spectra for AF-Li and B-Li metal surfaces; b) optical image of B-Li and AF-Li. The colored image indicates by (I) and (II) revealed the coverage of the protective layer on the lithium metal surface. (III) and (IV) are optical images for B-Li and AF-Li anode.

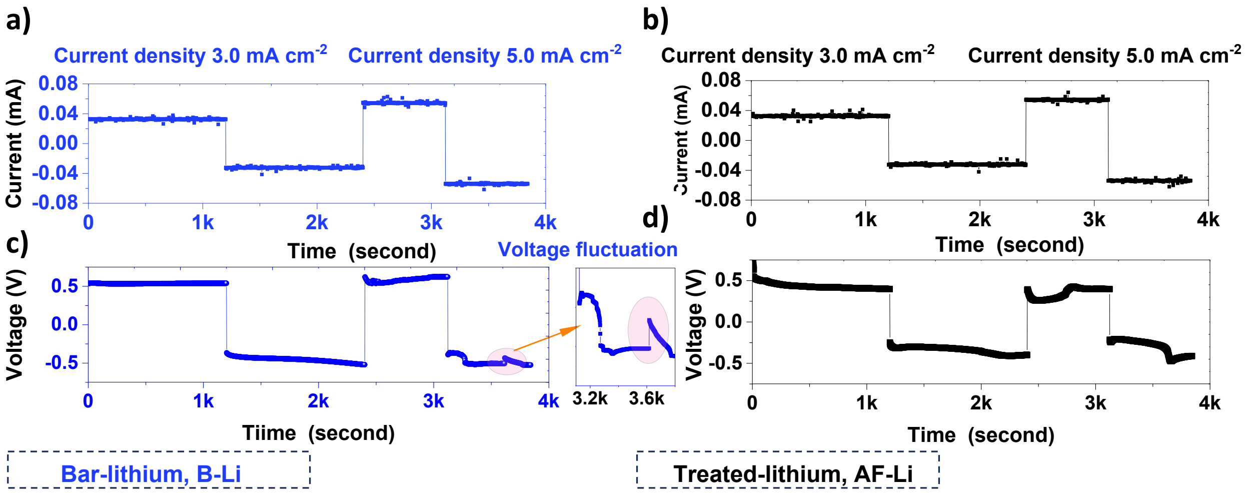

Figure S4. The I-t (current-time) and V-t (voltage-time) profiles are used for operand OM in under a symmetric cell system. a), b) are I-t and c), d) are V-t curves for B-Li (blue) and AF-Li (black) respectively.

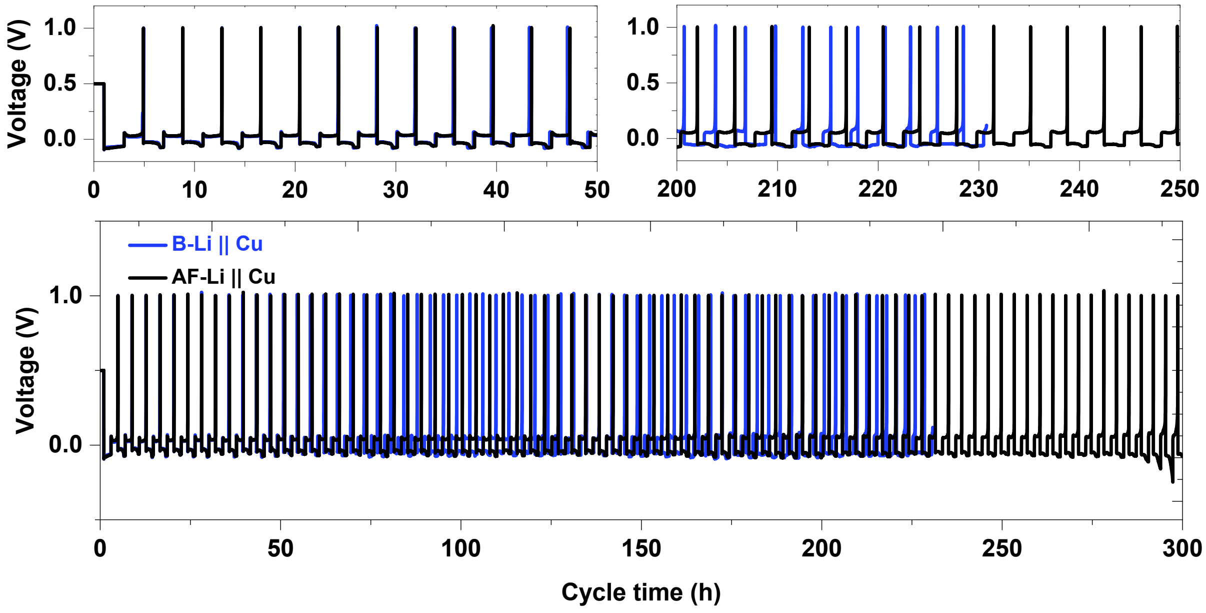

Figure S5. Voltage-time profile for half-cell performance of Li||Cu at 0.5 mA cm^{-2} (capacity 1 mA h cm^{-2}) with respect to B-Li and AF-Li lithium metal anode.

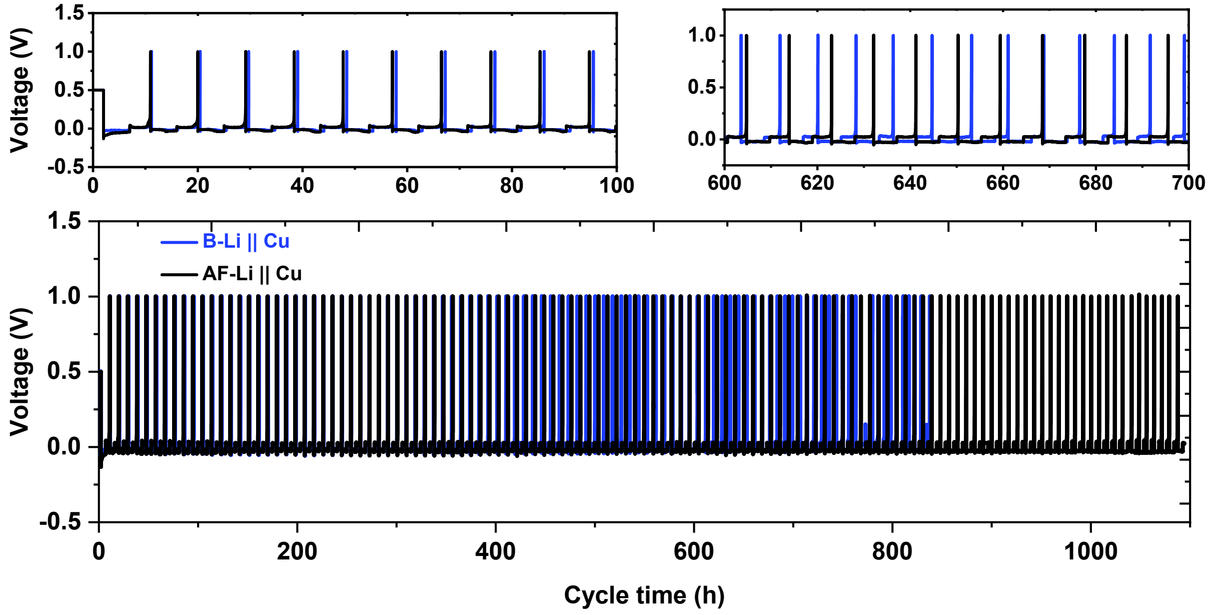

Figure S6. Voltage-time profile for half-cell performance of Li||Cu at 0.2 mA cm^{-2} (capacity 1 mA h cm^{-2}) with respect to B-Li and AF-Li lithium metal anode.

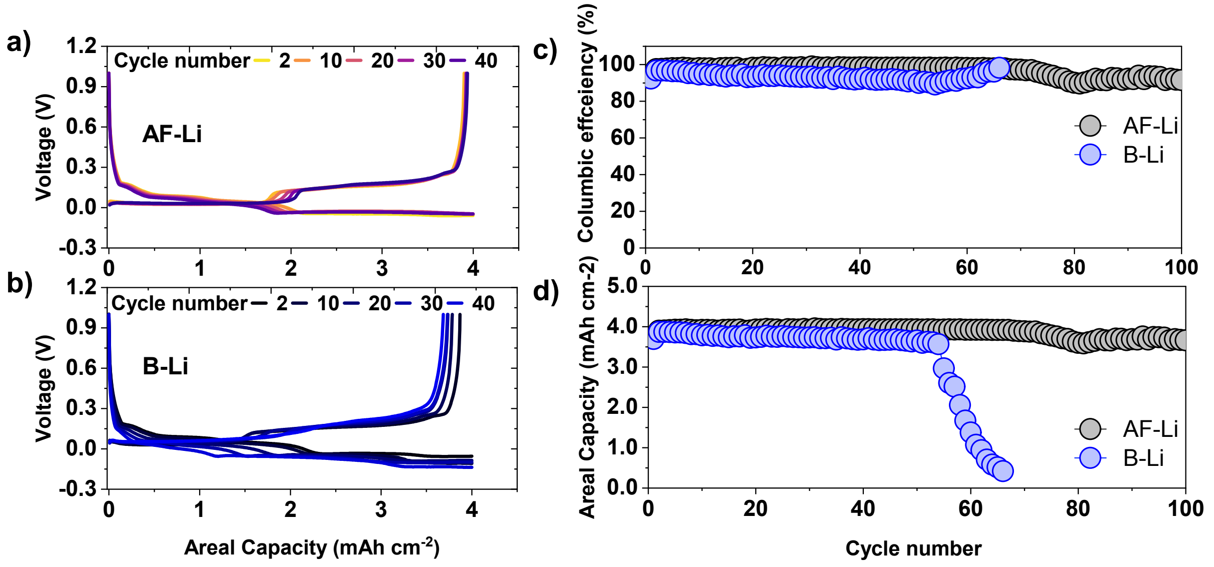

Figure S7. Half-cell, x-Li||MCMB (x = B-Li or AF-Li) electrochemical performance at 1 mA cm^{-2} currrent density. a) and b) voltage profile for B-Li and AF-Li metal anode with 4 mAh cm^{-2} areal capacity. c) coulombic efficiency of B-Li and AF-Li metal anodes and d) areal capacity (4 mAh cm^{-2}) for both lithium metal anode types.

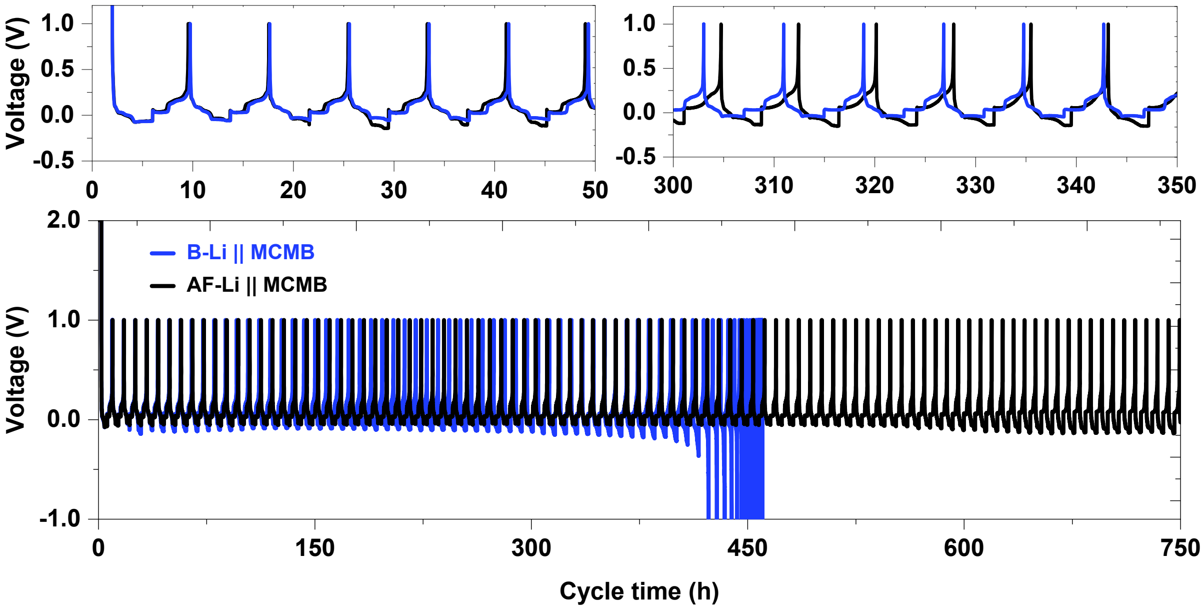

Figure S8. Voltage-time profile for half-cell performance of Li||MCMB at 1 mA cm^{-2} (capacity 4 mA h cm^{-2}) with respect to B-Li and AF-Li lithium metal anode.

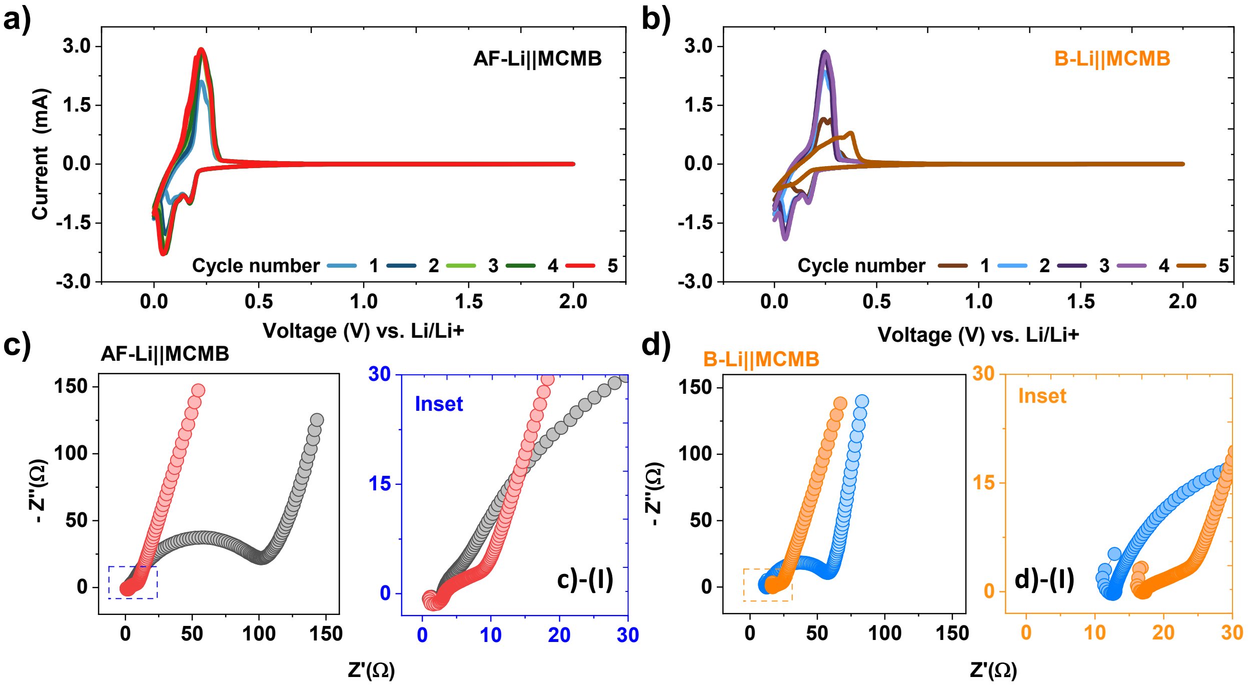

Figure S9. Cyclic voltammetric measurement integrated with EIS over cycling for Li||MCMB half-cell at 0.05 mV s^{-1} scan rate. a) and c) are CV and EIS plot for treated lithium metal AF-Li with inset of c)-(I) (blue) respectively. b) and d) corresponds to CV and EIS plot for B-lithium metal with an inset of d-(I) (orange).

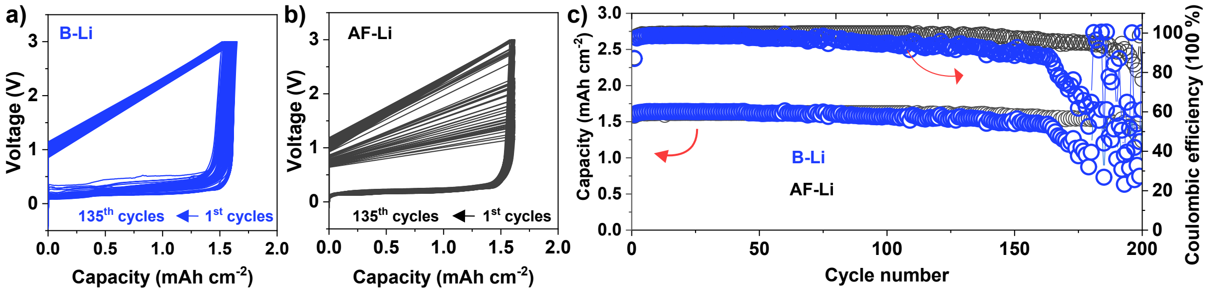

Figure S10. Half-cell performance of x-Li||MCMB (x = B-Li or AF-Li) at 2 mA cm^{-2} current density (capacity 2 mAh cm^{-2}). a) and b) voltage vs. capacity profile for B-Li and AF-Li metal anode for 135 cycles; c) comparison of capacity and coulombic efficiency of B-Li and AF-Li metal anodes long cycle.

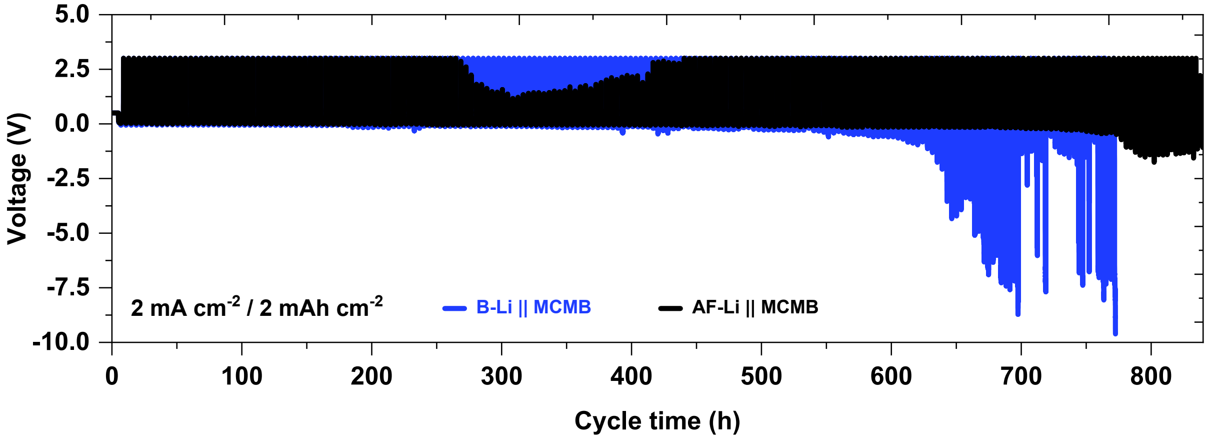

Figure S11. Voltage profile for x-Li||MCMB (x = B-Li or AF-Li) half-cell performance at 2 mA cm^{-2} current density (capacity 2 mAh cm^{-2}).

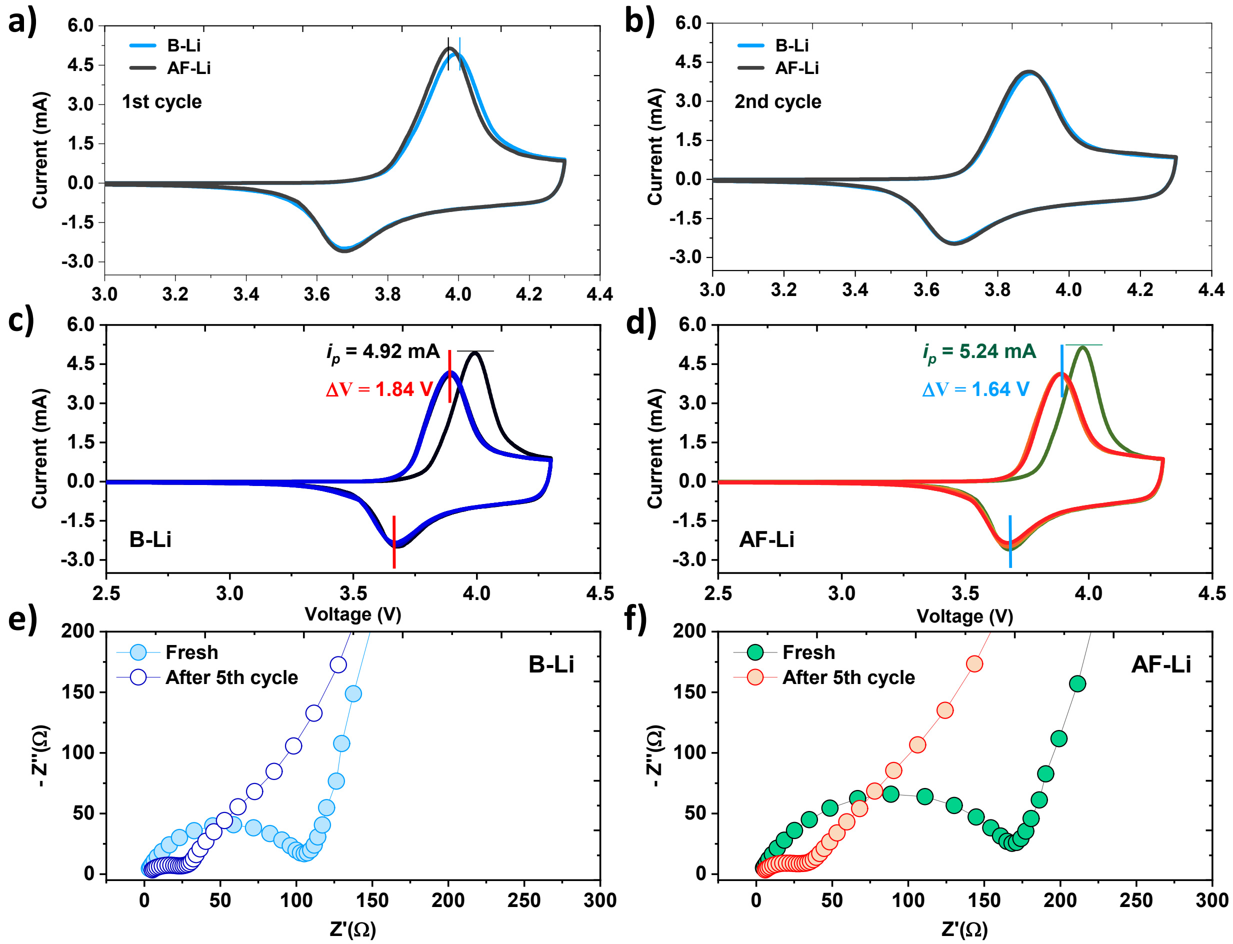

Figure S12. Cyclic voltammograms of NCM-523 ǁ x-Li (x = B or AF) operated at the scan rate of 0.1 mV s^{-1}. a) and b) illustrate a comparison of B-Li and AF-Li metal anode at 1st and 2nd cycles, while c) and d) show overpotential alteration for B-Li and AF-Li metal anode over cycling (up to 5th cycles) respectively. e) and f) indicate AC impedance measurement before and after 5th cycles for NCM-523 ǁ B-Li and NCM-523 ǁ AF-Li system respectively.

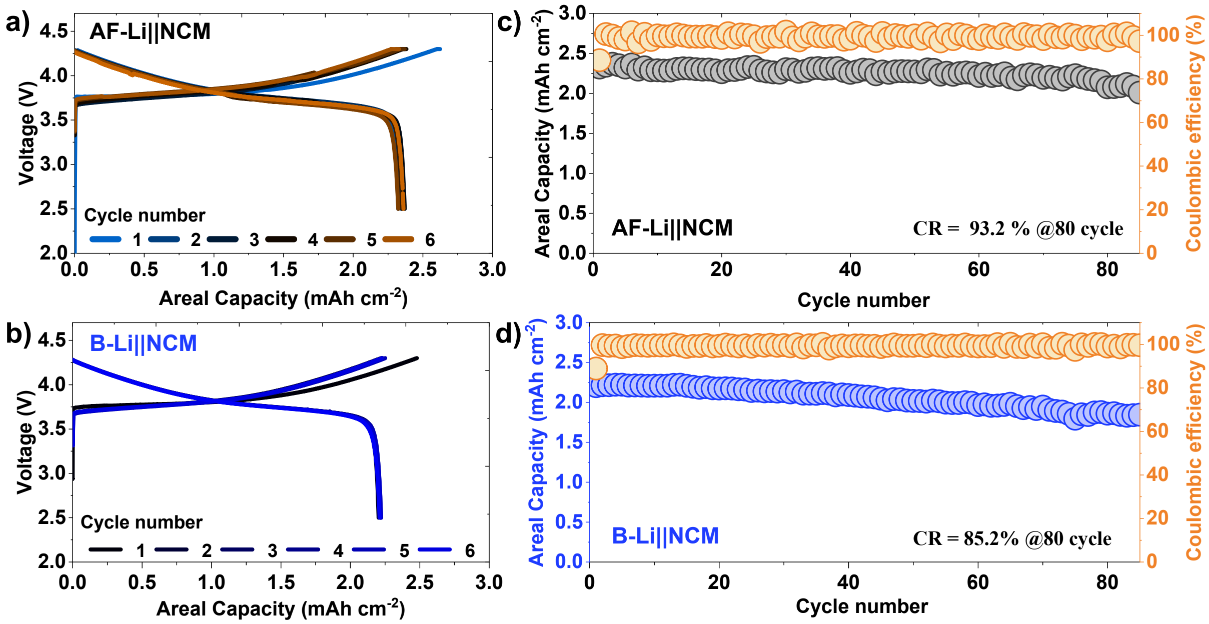

Figure S13. An electrochemical performance under NCM-111 ǁ x-Li (x = B or AF) cell configuration. Voltage profile for a) treated lithium metal anode, NCM-111 ǁ AF-Li B-Li metal anode; b) bare lithium metal anode, NCM-111 ǁ B-Li. c) and d) are cell performance for NCM-111 ǁ AF-Li and d) NCM-111 ǁ B-Li respectively.
